# Nursing AMS Forum

**DOI:** 10.1093/jacamr/dlz009

**Published:** 2019-04-12

## Abstract

**Graphical Abstract:**
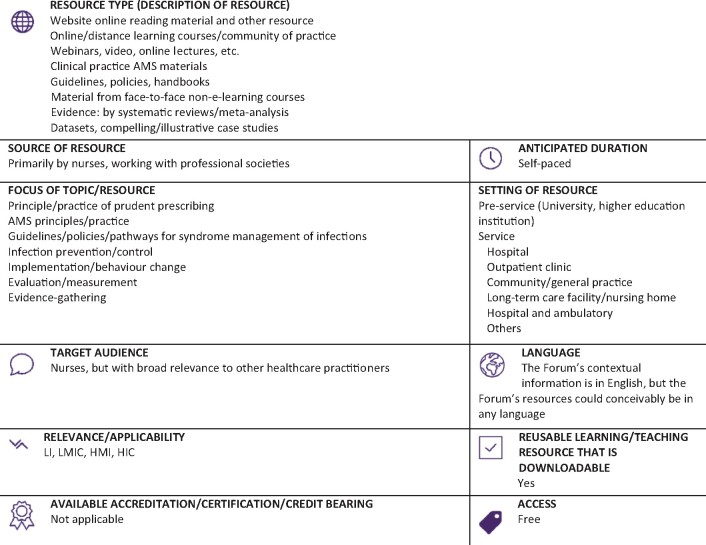

**Resource web link:** **http://www.nursing-ams-forum.co.uk** (Full classification scheme available at: http://bsac.org.uk/wp-content/uploads/2019/03/Educational-resource-review-classification-scheme.pdf)

**WHO region and country (World Bank):** Europe, UK (LI, LMIC, HMI, HIC)

## Peer review commentary

The Nursing AMS Forum is unique. It seeks to create a dynamic community of practice for an emerging—yet critically important—group of specialists. It aims to do this by providing two distinct, yet overlapping, sources of support: a repository for a range of antimicrobial resistance (AMR)/antimicrobial stewardship (AMS)-related resources and a topic-driven forum. Because it is a new venture (it was launched in November 2018 by BSAC) that relies upon the community itself to upload and utilise resources and engage in discussions, the likely extent of its impact is difficult to gauge, currently. Ultimately, its value will be determined by the frequency and quality of its use. As such, one of the biggest challenges will be to engage a significant number of nurses who, through self-regulation, will be prepared to identify the most relevant and up-to-date resources and discussion topics, while growing the Forum’s community by reaching out to colleagues across the world. As more and more resources are added to the Forum, it is important that the site maintains/develops a good search function with appropriate filters to help busy professionals find what they need, when they need it.

One of the difficulties, currently, is that the resource descriptors in the accordion menu are a bit short on detail. In the absence of more information, the user has to spend time investigating which resource is likely to yield what information. If this feature can be improved and if the general development can be sustained, the Forum it is likely to fulfil an important function that isn’t provided anywhere else. My only other caveat relates to the nature of the community itself. If its size remains modest and its nature remains homogeneous, it may give rise to the sort of cognitive bias which inhibits competition, innovation, and difference.

